# Clinical validation of the suppressive impact of letrozole on liver fibrosis in patients with breast cancer undergoing continuous letrozole administration: A retrospective study

**DOI:** 10.1371/journal.pone.0311930

**Published:** 2024-10-24

**Authors:** Kazuyoshi Ohkawa, Tasuku Nakabori, Kaori Mukai, Kazuhiro Kozumi, Makiko Urabe, Yugo Kai, Ryoji Takada, Kenji Ikezawa, Yuko Yamaguchi, Takuya Nagao, Hatsune Enomoto, Hidehisa Tachiki, Ayako Higuchi, Noriyuki Watanabe, Takahiro Nakayama

**Affiliations:** 1 Department of Hepatobiliary and Pancreatic Oncology, Osaka International Cancer Institute, Osaka, Japan; 2 Department of Clinical Research and Genetic Oncology, Osaka International Cancer Institute, Osaka, Japan; 3 Next-Generation Precision Medicine Research Center, Osaka International Cancer Institute, Osaka, Japan; 4 Research & Development Department, Towa Pharmaceutical Co., Ltd., Kadoma, Osaka, Japan; 5 Scientific Research and Business Development Department, Protosera, Inc., Settsu, Osaka, Japan; 6 Department of Breast and Endocrine Surgery, Osaka International Cancer Institute, Osaka, Japan; Shiraz University of Medical Sciences, ISLAMIC REPUBLIC OF IRAN

## Abstract

Treatment strategies for preventing liver fibrosis have not yet been established. Letrozole, widely used for breast cancer, has recently been reported to suppress liver fibrosis in murine models. Therefore, we aimed to validate the suppressive effects of letrozole on liver fibrosis in the clinical setting. From 2006 to 2020, 23 consecutive patients who received continuous letrozole treatment for 24 months or more and had a liver fibrosis marker FIB-4 index of ≥ 2.30, were included. Forty-three patients who underwent anastrozole treatment for 24 months or more and had a liver fibrosis marker FIB-4 index of ≥ 2.30, were also included as controls. The Fisher exact, chi-square, unpaired Student *t*, and paired Student *t* test were used to analyze the data. The patient characteristics were similar between the letrozole- and anastrozole-treated patient groups. Among the letrozole-treated patients, the mean FIB-4 index tended to decline during letrozole treatment; a significant decrease was observed at 18 and 24 months compared with the baseline values (p = 0.044 and p = 0.013). In addition, the mean aspartate aminotransferase-to-platelet ratio index (APRI) decreased during letrozole treatment; the values at 18 and 24 months were significantly lower than those at baseline (p = 0.024 and p = 0.026). In contrast, among anastrozole-treated patients, the mean FIB-4 index and APRI did not change during anastrozole treatment. When changes in the FIB-4 index were further examined in a limited number of patients with a FIB-4 index ≥ 2.67, a significant reduction in the FIB-4 index at 24 months compared with baseline was also observed in letrozole-treated patients (p = 0.023), but not in anastrozole-treated patients. In conclusion, our findings support a possible suppressive effect of letrozole on liver fibrosis in the clinical setting. Further studies are required to better understand the pharmacological effects of letrozole.

## Introduction

Chronic liver disease arises from various causes such as viral hepatitis, alcoholic and non-alcoholic steatotic liver disease, autoimmune liver disease and genetic disease, having become a significant global health concern [1]. There has been a recent shift in the main underlying etiologies of chronic liver disease from viral to steatotic [2–4]. In 2023, a nomenclature for steatotic liver disease was proposed [5]. In chronic liver disease, persistent inflammation causes the expansion of liver fibrosis, resulting in the development of liver cirrhosis [6]. Liver carcinogenesis occurs with the advancement of hepatic fibrosis [7]. Halting the progression of chronic liver disease requires the inhibition of liver fibrosis [8]. However, medical interventions to prevent liver fibrosis have yet to be established.

Letrozole, an aromatase inhibitor used as an anticancer drug in patients with breast cancer [9–11], has recently been reported by Sakai et al. to suppress liver fibrosis in a murine model [12]. In that report, 36 medicines were examined for their effects on gene expression in chimeric mice with humanized hepatocytes by microarray analysis with human gene probes. Among these, letrozole was selected for further analysis because it has a modifying effect on fibrosis-related gene expression in hepatocytes. Letrozole ameliorates liver fibrosis in carbon tetrachloride and methionine-choline deficient murine models. The mechanism involved inhibition of the *Yap-Ctgf* pathway, followed by a decrease in retinoic acid levels caused by suppression of *Hsd17b13* and activation of *Cyp26a1* [12]. However, the inhibitory effects of letrozole on hepatic fibrosis have not yet been assessed in humans. Such investigation is essential for advancing the clinical application of letrozole in the treatment of liver fibrosis.

Therefore, we aimed to validate the suppressive effects of letrozole on liver fibrosis in the clinical setting. Among patients with breast cancer who had undergone long-term letrozole treatment, those identified as having liver fibrosis on the basis of the liver fibrosis marker FIB-4 index [13] were included in this study. The inhibitory effect of letrozole on liver fibrosis was evaluated according to changes in liver fibrosis markers, the FIB-4 index and aspartate aminotransferase (AST)-to-platelet ratio index (APRI) [14] during letrozole treatment.

## Materials and methods

### Ethics statements

The present study was approved by the Institutional Review Board of Osaka International Cancer Institute (number: 20119–7) and conducted in accordance with the Declaration of Helsinki. Informed consent was obtained from patients using an opt-out form on the institution’s website.

### Study design and patients

**Fig 1:**
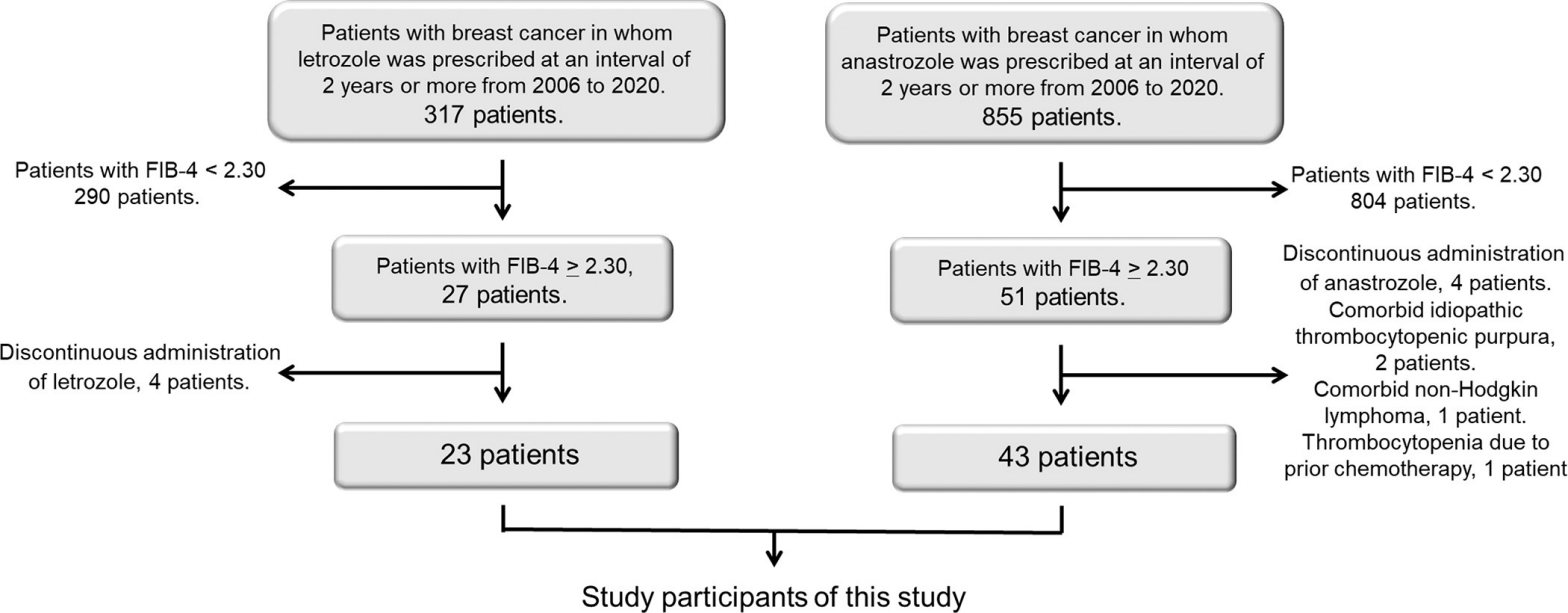
Flowchart of patient selection in this study.

Consecutive patients with breast cancer receiving continuous administration of the aromatase inhibitors letrozole and anastrozole for 24 months were included in this retrospective study. A flowchart of the patient selection process is shown in Fig 1. Between January 2006 and December 2020, 317 patients with breast cancer receiving letrozole at intervals ≥ 24 months were identified at the Osaka International Cancer Institute, among whom 27 exhibited a FIB-4 index of ≥ 2.30. Four patients were excluded owing to intermittent letrozole administration, resulting in the selection of 23 patients who received continuous letrozole treatment for 24 months or more. Likewise, among the 855 patients receiving anastrozole at intervals of ≥ 24 months during the same period, 51 had a FIB-4 index of ≥ 2.30. Following exclusion for various reasons, e.g., discontinuous anastrozole administration in 4 patients, comorbid idiopathic thrombocytopenic purpura in 2 patients, comorbid non-Hodgkin lymphoma in 1 patient, and thrombocytopenia due to prior chemotherapy in 1 patient, 43 patients who received continuous anastrozole treatment for 24 months or more were also selected as negative controls.

Baseline data on age, initially diagnosed disease or recurrence, prior treatment, prior hormone treatment, concurrent treatment, and underlying liver disease were collected for each patient. Platelet count, AST and alanine aminotransferase (ALT) levels were measured at baseline and at 6, 12, 18, and 24 months after letrozole and anastrozole treatment. The data utilized in the present study were accessed between September 2020 and August 2021 with as much care as possible to preserve anonymity of individual study participants during data collection.

### Liver fibrosis markers

Two indirect markers of liver fibrosis, FIB-4 index [13] and APRI [14], were calculated using the following formulas:

FIB-4 index = [age (years) × AST (U/L)] / [platelet count (10^9^/L) × ALT (U/L)^0.5^].

APRI = [(AST/upper limit of normal AST) × 100] / platelet count (10^9^/L).

An exhaustive review of medical records pertaining to each patient’s clinical course was conducted. Patients with fluctuating values of AST, ALT and platelet count owing to causes other than underlying chronic liver disease that affected the FIB-4 index and APRI were excluded from the study.

### Statistical analysis

The Fisher exact, chi-square, and unpaired Student *t* tests were used to compare patient characteristics and cancer treatment between the groups, as appropriate. The paired Student *t* test was used to compare liver fibrosis-related parameters before and after letrozole and anastrozole administration for each patient. All analyses were performed using SPSS (version 21.0; IBM Corp., Armonk, NY, USA). *P*-values <0.05 were considered statistically significant.

## Results

### Patient characteristics and cancer treatment

The characteristics of the patients and details of their breast cancer treatments are provided in S1 and S2 Tables, which present information on letrozole-treated patients (nos. 1–23) and anastrozole-treated patients (nos. 24–66). All patients included in the present study were postmenopausal females, and none of them were habitual alcohol drinkers. Liver metastatic lesions were not seen in any of the patients.

Among the 23 patients receiving letrozole treatment, the mean age was 70 ± 8 years. Twenty patients had early-stage breast cancer, whereas the remaining 3 had advanced breast cancer. Regarding underlying liver disease, 6 (26%) patients had metabolic dysfunction-associated steatotic liver disease (MASLD) [5], which was mainly determined by increased echogenicity of the liver and impaired visualization of intrahepatic vessels on B-mode ultrasonography. These patients also typically had obesity, type 2 diabetes mellitus, hypertension, or dyslipidemia. Chronic hepatitis C virus (HCV) infection was observed in 1 (4%) patient. Seven (30%) patients had chronic liver disease-like features characterized by dullness of the liver edge, a granular or nodular liver surface, and coarseness of the liver parenchymal echo pattern. In the remaining 9 (39%) patients, the diagnosis of chronic liver disease was uncertain. The mean APRI and FIB-4 index values were 0.65 ± 0.40 and 2.92 ± 0.69, respectively.

The mean age of the 43 patients who received anastrozole treatment was 71 ± 7 years. Forty-two patients had early-stage breast cancer, and 1 patient had advanced breast cancer. Regarding underlying liver disease, 8 (19%) patients had MASLD, 1 (2%) patient had chronic hepatitis B virus (HBV) infection, 3 (7%) patients had chronic HCV infection, and 11 (26%) patients showed chronic liver disease-like features. The diagnosis of liver disease was uncertain in the remaining 20 (47%) patients. The mean APRI and FIB-4 index values were 0.63 ± 0.26 and 2.94 ± 0.55, respectively.

When the clinical features and cancer treatments were compared between patients who underwent treatment with letrozole and anastrozole (Table 1), age, breast cancer status, prior treatment, prior hormone treatment, concurrent treatment, underlying liver disease, APRI, and FIB-4 index did not differ between the groups.

**Table 1:** Clinical features and cancer treatment in patients with breast cancer receiving letrozole and anastrozole.

Characteristics	Patients receiving	Patients receiving	*p*-value
	letrozole treatment	anastrozole treatment	
	(n=23)	(n=43)	
Age (years)	70 ± 8^b^	71 ± 7	NS
Breast cancer status (early/advanced)	20/3	42/1	NS
Prior treatment			
Surgery	20 (87%)	43 (100%)	NS
Chemotherapy	6 (26%)	7 (16%)	NS
Radiotherapy	11 (48%)	18 (42%)^c^	NS
HER2-targeting drug	2 (9%)	3 (7%)	NS
Prior hormone treatment			
Tamoxifen	4 (17%)	3 (7%)	NS
Exemestane	1 (4%)	0 (0%)	NS
Anastrozole	2 (9%)	ー	ー
Letrozole	ー	1 (2%)	ー
Concurrent treament			
Chemotherapy^a^	1 (4%)	3 (7%)	NS
CDK4/6 inhibitor	1 (4%)	0 (0%)	NS
HER2-targeting drug	0 (0%)	4 (9%)	NS
Underlying liver disease			
MASLD	6 (26%)	8 (19%)	NS
Chronic hepatitis B	0 (0%)	1 (2%)	NS
Chronic hepatitis C	1 (4%)	3 (7%)	NS
Chronic liver disease-like	7 (30%)	11 (26%)	NS
Uncertain	9 (39%)	20 (47%)	NS
APRI	0.65 ± 0.40	0.63 ± 0.26	NS
FIB-4 index	2.92 ± 0.69	2.94 ± 0.55	NS

HER2, human epidermal growth factor receptor 2.

CDK4/6, cyclin-dependent kinase 4/6.

NS, not significant.

^a^Tegaful-uracil was used for chemotherapy concurrent with letrozole/anastrozole.

^b^Values are expressed as mean ± standard deviation.

^c^Radiothrerapy was carried out concurrently with anastrozole administration in eight patients.

### Changes in the liver fibrosis markers during letrozole and anastrozole treatment

**Fig 2:**
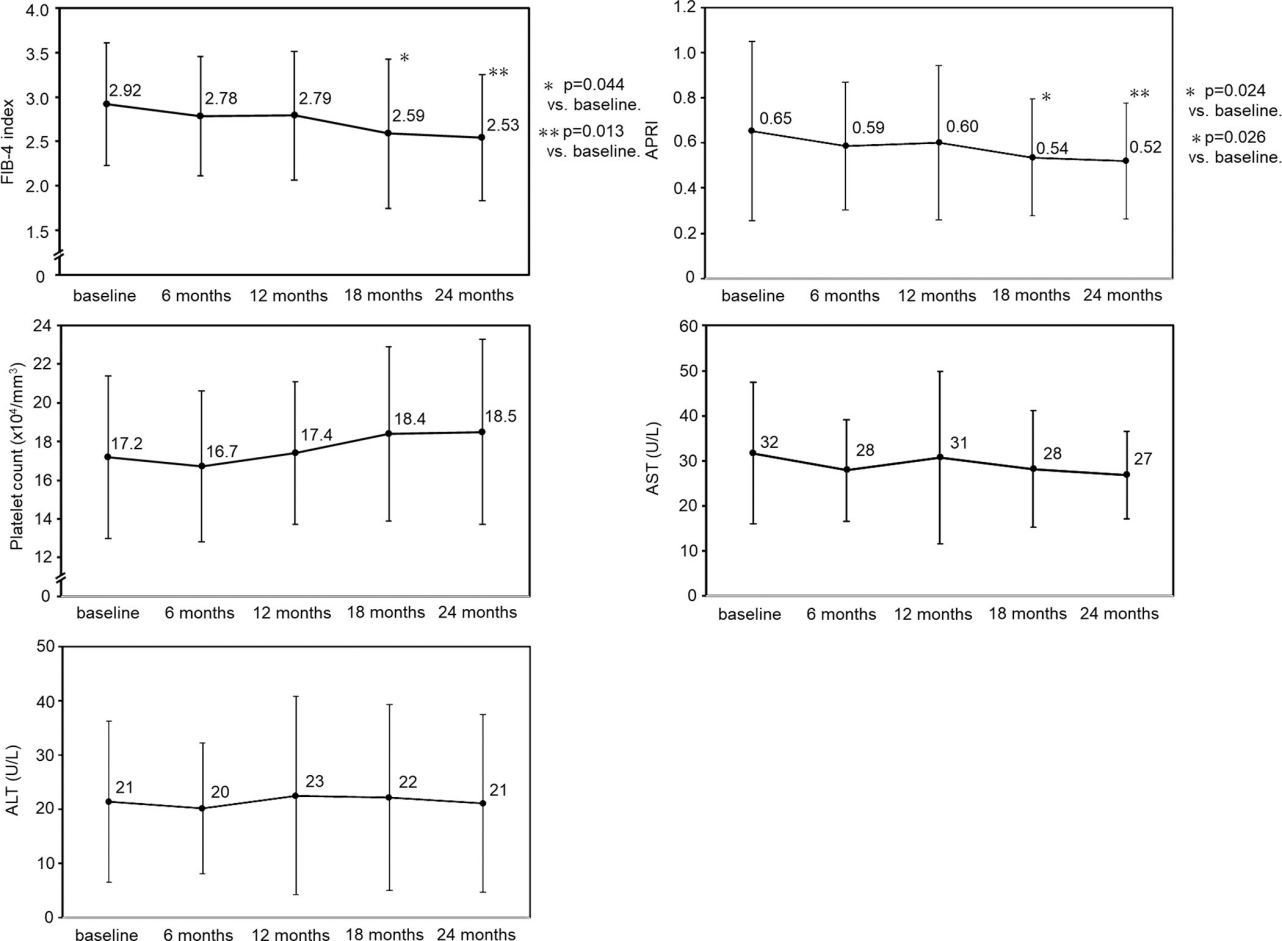
Changes in the liver fibrosis-related markers in breast cancer patients with a FIB-4 index of >2.30 during letrozole treatment.

**Fig 3:**
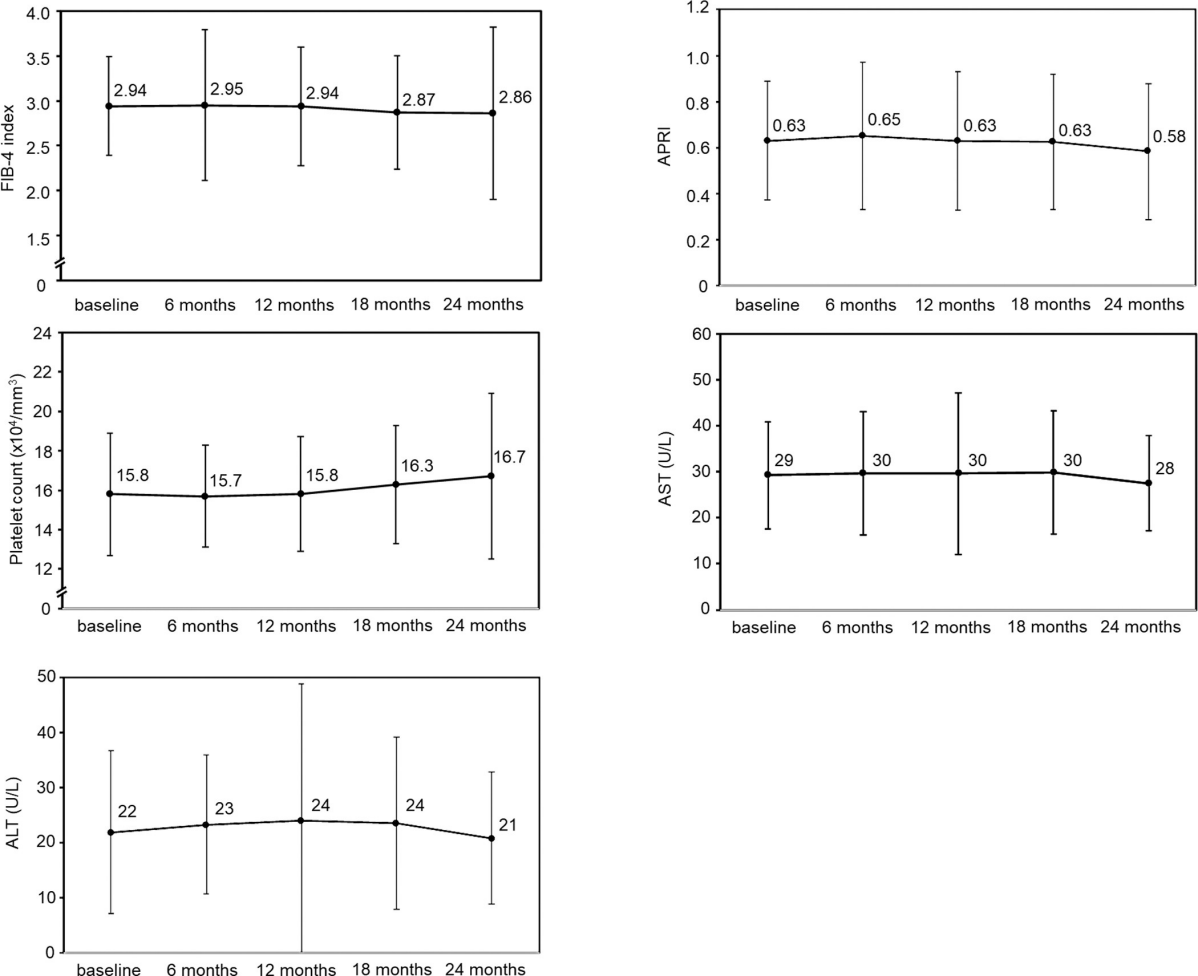
Changes in the liver fibrosis-related markers in breast cancer patients with a FIB-4 index of >2.30 during anastrozole treatment.

Next, changes in liver fibrosis markers were investigated during letrozole and anastrozole treatment in breast cancer patients with liver fibrosis, as judged by a FIB-4 index of ≥ 2.30 at baseline. The raw data for the analysis are provided in S1 Data. Among the patients who received letrozole administration (Fig 2), the mean FIB-4 index values were 2.92 ± 0.69, 2.78 ± 0.67, 2.79 ± 0.72, 2.59 ± 0.84 and 2.53 ± 0.71 at baseline, 6, 12, 18 and 24 months, respectively. A significant decrease in mean FIB-4 index values was observed at 18 and 24 months (p = 0.044 and p = 0.013, respectively) compared with baseline. The mean APRI values were 0.65 ± 0.40, 0.59 ± 0.28, 0.60 ± 0.34, 0.54 ± 0.26 and 0.52 ± 0.26 at baseline, 6, 12, 18 and 24 months, respectively; the values at 18 and 24 months were significantly lower than those at baseline (p = 0.024 and p = 0.026, respectively). Platelet count, AST and ALT values did not change significantly after letrozole treatment. However, when the patients receiving anastrozole administration were examined (Fig 3), the respective mean FIB-4 index values were 2.94 ± 0.55, 2.95 ± 0.84, 2.94 ± 0.66, 2.87 ± 0.63 and 2.86 ± 0.96, and the respective mean APRI values were 0.63 ± 0.26, 0.65 ± 0.32, 0.63 ± 0.30, 0.63 ± 0.29 and 0.58 ± 0.30 at baseline, 6, 12, 18 and 24 months. No significant changes in the FIB-4 index or APRI were observed during anastrozole treatment. In addition, the platelet, AST, and ALT values did not change during anastrozole treatment.

**Fig 4:**
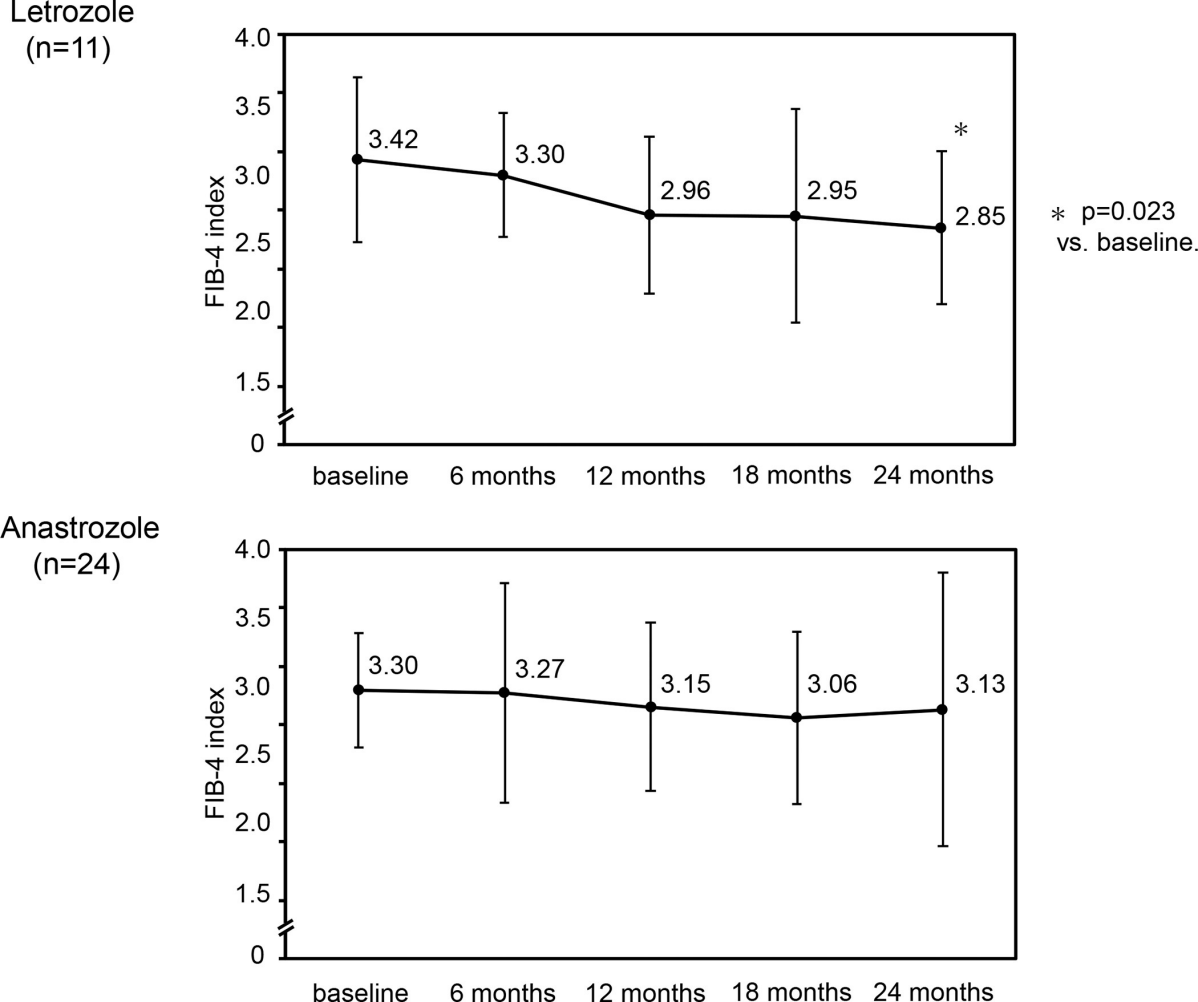
Changes in the FIB-4 index in breast cancer patients with a FIB-4 index of ≥ 2.67 during letrozole and anastrozole treatment.

Changes in the FIB-4 index were further investigated in the limited number of breast cancer patients with a FIB-4 index of ≥ 2.67 (11 letrozole-treated and 24 anastrozole-treated patients), which was the generally used cut-off value [15–17]. As shown in Fig 4, a significant reduction in the FIB-4 index at 24 months compared with that at baseline was observed in letrozole-treated patients (p = 0.023) but not in anastrozole-treated patients.

### Clinical course of a representative patient showing a suppressive effect of letrozole on liver fibrosis

**Fig 5:**
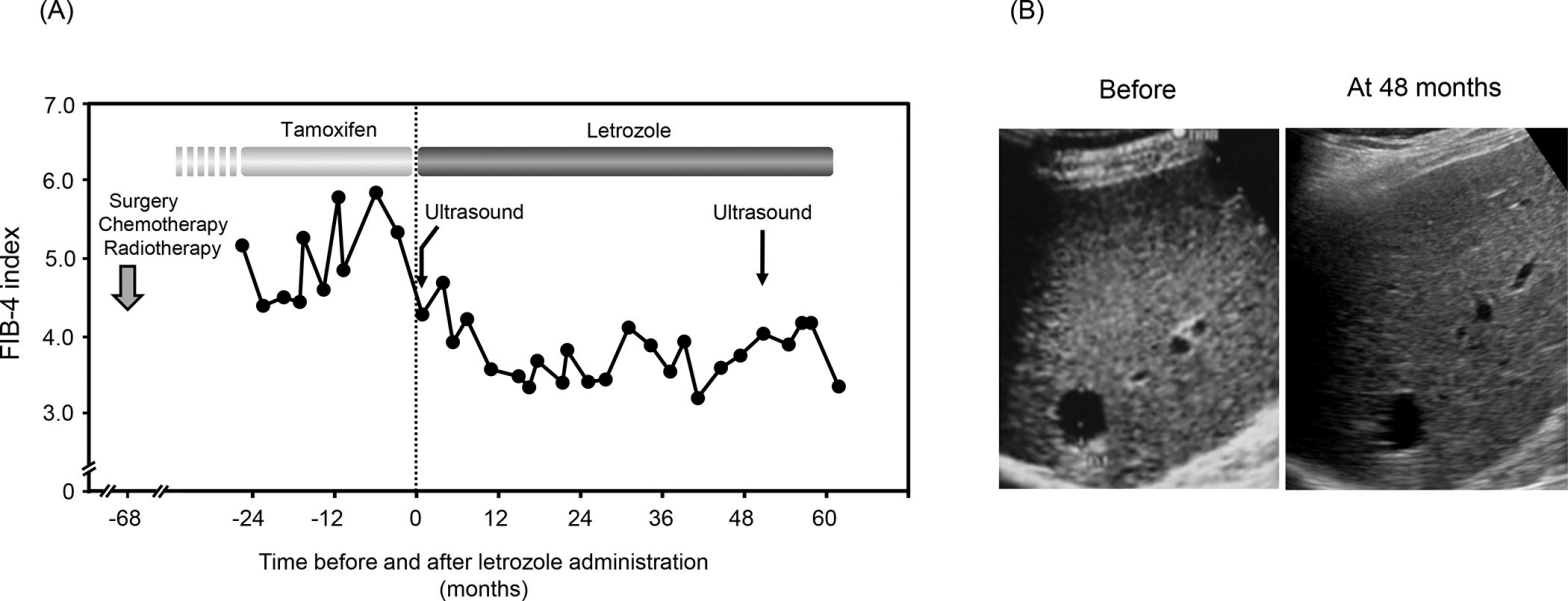
Clinical course of patient no. **1.** (A) Patient 1 underwent letrozole treatment and showed a possible suppressive effect of letrozole on liver fibrosis. (B) Ultrasonograms at baseline and 48 months of letrozole treatment.

Fig 5A shows the clinical course of patient no. 1, who underwent long-term letrozole administration and showed a possible suppressive effect on liver fibrosis. She had a chronic HCV infection and did not receive antiviral treatment during follow-up. For breast cancer, partial mastectomy with axillary lymph node dissection; chemotherapy with cyclophosphamide, methotrexate and fluorouracil; and radiotherapy were performed, followed by tamoxifen administration. The FIB-4 index tended to decrease during tamoxifen treatment, and then the patient was switched to letrozole treatment. The FIB-4 index tended to decrease after long-term letrozole administration. Fig 5B shows the ultrasonograms at baseline and 48 months of letrozole treatment. The irregularity of the liver surface and the coarseness of the liver parenchymal echo improved considerably at 48 months (right) of letrozole treatment compared with that at baseline (left), indicating a possible improvement in liver fibrosis.

## Discussion

In patients with chronic liver disease, disease progression to cirrhosis and hepatocellular carcinoma is often observed accompanied by advanced liver fibrosis [6, 7]. Prevention of liver fibrosis is one of the important clinical challenges [8]. However, such treatment strategies have not been fully established. Letrozole has recently been reported to have an inhibitory effect on liver fibrosis in a murine model [12]. In the present study, we aimed to validate the suppressive effects of letrozole on liver fibrosis. In letrozole-treated breast cancer patients with liver fibrosis, as judged by the FIB-4 index [13], changes in liver fibrosis markers were examined during long-term letrozole administration. Our results showed that both liver fibrosis markers, the FIB-4 index and APRI, significantly decreased during continuous letrozole treatment for up to 24 months. In contrast, liver fibrosis markers did not decrease in patients treated with anastrozole, another aromatase inhibitor used as the negative control. This tendency was also observed in patients with a FIB-4 index of ≥ 2.67 [15–17], who were more likely to have progressive liver fibrosis. In one patient (patient 1), the FIB-4 index tended to increase during tamoxifen administration but tended to decrease after switching to letrozole treatment. Repeated abdominal ultrasonography revealed an improvement in liver fibrosis. These results indicate that letrozole may have a suppressive effect on liver fibrosis not only in a murine model but also in human clinical practice.

In the present study, letrozole- and anastrozole-treated patients were retrospectively compared. There may have been a potential bias in the two patient groups because the drug for hormone treatment was selected according to the attending doctor’s judgement. However, clinical features and cancer treatment did not differ significantly between the two groups. Thus, it is plausible that this bias may not have significantly affected the study outcomes.

The main drawback of our study was the lack of direct evaluation of liver fibrosis. Only the FIB-4 index [13] and APRI [14] have been used to evaluate liver fibrosis, which appear to be inadequate. Liver fibrosis is most accurately assessed histologically using repeated liver biopsy, which is considered as the gold standard [18]. The next best diagnostic modality is the measurement of liver stiffness by means of ultrasonography or magnetic resonance elastography [19–21]. The letrozole-mediated suppressive effects on liver fibrosis need to be verified by further studies using more accurate modalities to assess liver fibrosis.

The cut-off value of 2.30 for the FIB-4 index used in this study is uncommon. It was examined at every 0.1, and a value of 2.30 was merely applied as the most appropriate cut-off value in the present study. Instead, the more frequently used cut-off value of 2.67 [15–17] was also examined. Although the number of patients was smaller, a tendency toward lower FIB-4 values during letrozole treatment was observed. This suggests that setting the cut-off value for the FIB-4 index may not have substantially influenced the study results.

Letrozole and anastrozole are aromatase inhibitors used to treat breast cancer [22, 23]. In our study, the inhibitory effect on liver fibrosis, as judged by the decrease in the FIB-4 index and APRI values, was exhibited by letrozole but not by anastrozole. Thus, letrozole may exert an inhibitory effect on liver fibrosis via structural regions other than the active center of the aromatase inhibitor. However, further studies are required to investigate this further.

In a previous study, Sakai et al. [12]. demonstrated the inhibitory effect of letrozole on liver fibrosis in a murine model; such an effect was observed only in males. In contrast, in the current clinical investigation, all patients were postmenopausal females, which may potentially offer a closer approximation of the male condition in the murine model. Understanding the influence of sex differences on the efficacy of letrozole is needed for its prospective clinical application in the inhibition of liver fibrosis.

When employing aromatase inhibitors in the treatment of postmenopausal breast cancer, multiple drugs, including letrozole and anastrozole, are available as therapeutic options. Our results suggest that letrozole may be a practical choice among these aromatase inhibitors for patients with chronic liver disease and significant liver fibrosis. This may represent a main clinical implication of our study at present.

In conclusion, our findings indicate that letrozole may have a suppressive effect on liver fibrosis in the clinical setting. In view of the limitations of our study, including its single-center, retrospective design and the relatively small sample size, further detailed prospective studies with larger numbers of patients are required to better understand the pharmacological mechanism of letrozole-mediated inhibition of liver fibrosis. Our study could potentially serve as an initial step towards the development of a new class of inhibitors targeting liver fibrosis.

## Supporting information

S1 Table Clinical features of and cancer treatment in 23 patients with breast cancer with FIB-4 index ≥ 2.30 who underwent letrozole treatment. (DOCX)

S2 Table Clinical features of and cancer treatment in 43 patients with breast cancer with FIB-4 index ≥ 2.30 who underwent anastrozole treatment. (DOCX)

S1 Data (XLSX)
