# Supplementary material for: Clinical validation of the suppressive impact of letrozole on liver fibrosis in patients with breast cancer undergoing continuous letrozole administration: A retrospective study
Source: PLoS One. 2024 Oct 24;19(10):e0311930. doi: 10.1371/journal.pone.0311930 (PMC11500940; doi:10.1371/journal.pone.0311930)
Supplement: Supplementary file 1 [file pone.0311930.s001.docx]

Supplementary Table 1. Clinical features and cancer treatment in 23 patients with breast cancer with FIB-4 index ≥ 2.30 who underwent letrozole treatment.

Patient no.	Age (years)	Breast cancer status	Initial diagnosis/ recurrence	Treatment prior to letrozole[a]	Drugs used for prior treatment	Hormone treatment prior to letrozole[a]	Combination drug with letrozole	Liver disease[b]	APRI	FIB-4 index
1	60	early	initial diagnosis	surgery, chemotherapy, radiotherapy	cyclophosphamide, methotrexate fluorouracil	tamoxifen	none	chronoic hepatitis C	2.08	5.34
2	73	early	initial diagnosis	surgery, chemotherapy	cyclophosphamide, methotrexate fluorouracil	tamoxifen	none	MASLD	0.58	2.97
3	68	early	initial diagnosis	surgery, radiotherapy			none	chronic liver disease-like	0.48	2.43
4	74	early	initial diagnosis	surgery			none	uncertain	1.28	3.53
5	59	early	initial diagnosis	surgery			none	chronic liver disease-like	0.44	2.35
6	68	early	initial diagnosis	surgery			none	MASLD	0.40	2.48
7	64	early	initial diagnosis	surgery, chemotherapy	fluorouracil, epirubicin cyclophosphamide		none	MASLD	1.16	3.82
8	67	early	initial diagnosis	surgery, radiotherapy			none	chronic liver disease-like	0.37	2.49
9	67	early	initial diagnosis	chemotherapy, surgery, radiotherapy	capecitabine, cyclophosphamide		none	MASLD	0.67	3.10
10	72	early	initial diagnosis	surgery			none	MASLD	0.82	2.55
11	76	early	initial diagnosis	surgery, radiotherapy			none	chronic liver disease-like	0.38	2.31
12	69	early	initial diagnosis	surgery, chemotherapy, trastuzumab	docetaxel, cyclophosphamide trastuzumab	anastrozole	none	uncertain	0.49	2.47
13	58	early	initial diagnosis	surgery, radiotherapy, chemotherapy, trastuzumab	fluorouracil, epirubicin cyclophosphamide, docetaxel trastuzumab	tamoxifen	none	uncertain	0.83	2.74
14	76	early	initial diagnosis	surgery, radiotherapy			none	uncertain	0.53	3.24
15	70	early	initial diagnosis	surgery, radiotherapy			none	uncertain	0.43	2.62
16	75	early	initial diagnosis	surgery, radiotherapy			none	chronic liver disease-like	0.35	2.65
17	72	early	initial diagnosis	surgery, chemotherapy	docetaxel, cyclophosphamide		none	uncertain	0.40	2.30
18	84	early	recurrence (local)	surgery			none	chronic liver disease-like	0.50	3.33
19	56	early	recurrence (local)	surgery			none	MASLD	0.86	2.51
20	78	early	recurrence (local)	surgery, radiotherapy		anastrozole, exemestane	none	uncertain	0.48	2.98
21	83	advanced	recurrence (lymph node)	none		tamoxifen	none	chronic liver disease-like	0.51	3.51
22	83	advanced	recurrence (distant)	none			tegaful-uracil	uncertain	0.52	3.12
23	60	advanced	initial diagnosis	radiotherapy (for bone metastasis)			palbociclib	uncertain	0.47	2.46

[a]Content of the initial treatment was excluded in the case of patients with recurrent breast cancer.

[b]Liver disease was diagnosed based on ultrasound image and laboratory data.
