# Supplementary material for: Clinical validation of the suppressive impact of letrozole on liver fibrosis in patients with breast cancer undergoing continuous letrozole administration: A retrospective study
Source: PLoS One. 2024 Oct 24;19(10):e0311930. doi: 10.1371/journal.pone.0311930 (PMC11500940; doi:10.1371/journal.pone.0311930)
Supplement: Supplementary file 2 [file pone.0311930.s002.docx]

Supplementary Table 2. Clinical features and cancer treatment in 43 patients with breast cancer with FIB-4 index $\geq$ 2.30 who underwent anastrozole treatment.

Patient no.	Age (years)	Breast cancer status	Initial diagnosis/ recurrence	Treatment prior to anastrozole[a]	Drugs used for prior treatment	Hormone treatment prior to anastrozole[a]	Combination drug with anastrozole	Liver disease[b]	APRI	FIB-4 index
24	61	early	initial diagnosis	surgery, radiotherapy			none	uncertain	0.66	3.49
25	64	early	initial diagnosis	surgery, chemotherapy, radiotherapy	fluorouracil, epirubicin cyclophosphamide		none	uncertain	0.69	2.76
26	80	early	initial diagnosis	surgery, radiotherapy			none	chronic liver disease-like	0.43	2.41
27	68	early	initial diagnosis	surgery			none	MASLD	0.98	2.75
28	65	early	initial diagnosis	surgery, radiotherapy			none	MASLD	1.56	3.41
29	75	early	initial diagnosis	surgery, radiotherapy[c]			none	uncertain	0.46	2.48
30	66	early	initial diagnosis	surgery		tamoxifen	none	uncertain	0.53	2.52
31	52	early	initial diagnosis	surgery			none	uncertain	0.34	2.36
32	59	early	initial diagnosis	surgery, chemotherapy	fluorouracil, epirubicin cyclophosphamide	letrozole	none	uncertain	0.59	2.54
33	75	early	initial diagnosis	surgery			tegaful-uracil	chronoic hepatitis B	0.46	3.23
34	82	early	initial diagnosis	surgery, chemotherapy, radiotherapy[c] trastuzumab	epirubicin, cyclophosphamide docetaxel, trastuzumab		trastuzumab	chronic liver disease-like	0.56	3.82
35	78	early	initial diagnosis	surgery			trastuzumab	uncertain	0.38	2.66
36	66	early	initial diagnosis	surgery, radiotherapy		tamoxifen	none	chronic liver disease-like	0.42	2.41
37	73	early	initial diagnosis	surgery, radiotherapy			none	MASLD	0.70	2.36
38	76	early	initial diagnosis	surgery			none	chronic liver disease-like	0.60	3.63
39	72	early	initial diagnosis	surgery			none	chronic liver disease-like	0.48	2.57
40	64	early	initial diagnosis	surgery, radiotherapy			none	chronoic hepatitis C	1.28	3.17
41	71	early	initial diagnosis	surgery, radiotherapy[c]			none	MASLD	0.57	2.54
42	67	early	initial diagnosis	surgery, chemotherapy	fluorouracil, epirubicin cyclophosphamide		trastuzumab	chronic liver disease-like	0.90	3.93
43	78	early	initial diagnosis	surgery			none	chronic liver disease-like	0.34	2.42
44	73	early	initial diagnosis	surgery			none	uncertain	0.56	2.55
45	66	early	initial diagnosis	surgery			none	uncertain	0.61	3.03
46	70	early	initial diagnosis	surgery			none	uncertain	0.49	2.51
47	77	early	initial diagnosis	surgery			none	uncertain	0.53	3.07
48	68	early	initial diagnosis	surgery, radiotherapy[c]			none	chronoic hepatitis C	0.74	2.95
49	69	early	initial diagnosis	surgery			none	chronic liver disease-like	0.43	2.48
50	79	early	initial diagnosis	surgery			none	MASLD	0.57	2.72
51	67	early	initial diagnosis	surgery			none	chronic liver disease-like	0.66	3.65
52	74	early	initial diagnosis	surgery		tamoxifen	none	uncertain	0.50	2.93
53	71	early	initial diagnosis	surgery, chemotherapy trastuzumab	fluorouracil, epirubicin cyclophosphamide paclitaxel, trastuzumab		trastuzumab	uncertain	0.51	3.59
54	75	early	initial diagnosis	surgery, radiotherapy[c]			none	MASLD	1.19	4.46
55	76	early	initial diagnosis	surgery			none	uncertain	0.61	2.82
56	76	early	initial diagnosis	surgery, radiotherapy[c]			none	chronoic hepatitis C	0.55	2.87
57	80	early	initial diagnosis	surgery			none	uncertain	0.44	2.35
58	75	early	initial diagnosis	surgery, radiotherapy[c]			none	MASLD	1.05	3.77
59	69	early	initial diagnosis	surgery, radiotherapy			tegaful-uracil	uncertain	0.44	2.51
60	87	early	initial diagnosis	surgery			none	uncertain	0.36	2.36
61	68	early	initial diagnosis	surgery, radiotherapy[c]			none	uncertain	0.48	2.69
62	80	early	initial diagnosis	surgery			none	uncertain	0.71	3.36
63	63	early	initial diagnosis	surgery, radiotherapy			none	uncertain	0.56	2.65
64	85	early	initial diagnosis	surgery			tegaful-uracil	chronic liver disease-like	0.89	4.15
65	67	early	recurrence (local)	surgery, chemotherapy	fluorouracil, epirubicin cyclophosphamide, docetaxel		none	chronic liver disease-like	0.65	2.62
66	61	advanced	initial diagnosis	chemotherapy, surgery, radiotherapy trastuzumab	fluorouracil, epirubicin cyclophosphamide docetaxel, trastuzumab		none	MASLD	0.70	3.04

[a]Content of the initial treatment was excluded in the case of patients with recurrent breast cancer.

[b]Liver disease was diagnosed by ultrasound image and laboratory data.

[c]Radiotherapy was carried out concurrently with anastrozole administration.
